# The Experience Elicited by Hallucinogens Presents the Highest Similarity to Dreaming within a Large Database of Psychoactive Substance Reports

**DOI:** 10.3389/fnins.2018.00007

**Published:** 2018-01-22

**Authors:** Camila Sanz, Federico Zamberlan, Earth Erowid, Fire Erowid, Enzo Tagliazucchi

**Affiliations:** ^1^Departamento de Física, Universidad de Buenos Aires, Buenos Aires, Argentina; ^2^Erowid Center, Grass Valley, CA, United States; ^3^Brain and Spine Institute, Paris, France

**Keywords:** dreams, psychedelics, dissociatives, deliriants, hallucinogens, phenomenology, consciousness

## Abstract

Ever since the modern rediscovery of psychedelic substances by Western society, several authors have independently proposed that their effects bear a high resemblance to the dreams and dreamlike experiences occurring naturally during the sleep-wake cycle. Recent studies in humans have provided neurophysiological evidence supporting this hypothesis. However, a rigorous comparative analysis of the phenomenology (“what it feels like” to experience these states) is currently lacking. We investigated the semantic similarity between a large number of subjective reports of psychoactive substances and reports of high/low lucidity dreams, and found that the highest-ranking substance in terms of the similarity to high lucidity dreams was the serotonergic psychedelic lysergic acid diethylamide (LSD), whereas the highest-ranking in terms of the similarity to dreams of low lucidity were plants of the *Datura* genus, rich in deliriant tropane alkaloids. Conversely, sedatives, stimulants, antipsychotics, and antidepressants comprised most of the lowest-ranking substances. An analysis of the most frequent words in the subjective reports of dreams and hallucinogens revealed that terms associated with perception (“see,” “visual,” “face,” “reality,” “color”), emotion (“fear”), setting (“outside,” “inside,” “street,” “front,” “behind”) and relatives (“mom,” “dad,” “brother,” “parent,” “family”) were the most prevalent across both experiences. In summary, we applied novel quantitative analyses to a large volume of empirical data to confirm the hypothesis that, among all psychoactive substances, hallucinogen drugs elicit experiences with the highest semantic similarity to those of dreams. Our results and the associated methodological developments open the way to study the comparative phenomenology of different altered states of consciousness and its relationship with non-invasive measurements of brain physiology.

## Introduction

“*The indolic alkaloids psilocybine and psilocine are the main hallucinogenic principles of the sacred mushrooms (…). The mushrooms cause both visual and auditory hallucinations, with the dreamlike state becoming reality”* (Schultes and Hofmann, 1979).

Our everyday experience of wakefulness is only one among many different states or modes of consciousness that are available to us. This experience can be diminished or even disappear during states such as deep dreamless sleep, under anesthesia, coma or in the vegetative state (Boly and Seth, 2012; Hobson, 2017). However, other brain states are characterized by more subtle modifications to the contents of consciousness. The most frequent of such modifications appear in the form of vivid dreams during the rapid eye movement (REM) phase of healthy human sleep. Dreams are characterized by vivid multimodal imagery (sometimes construed as realistic “hallucinations”), loss of the sense of agency and volitional control, dissociation between the first-person point of view and the bodily self of the dreamer, suppressed metacognitive function and heightened emotional reactivity (Hobson, 2009; Nir and Tononi, 2010). Dreams during which the dreamer is aware of experiencing a dream instead of wakefulness and does not identify with the “dreaming self” but with the everyday waking self are usually termed “lucid dreams” (La Berge et al., 1981; Voss et al., 2009).

Conscious states sharing some of these features with dreaming occur as a consequence of neuropsychiatric disorders (Hobson, 1999; Scarone et al., 2007), can be induced by psychoactive substances (Kraehenmann, 2017) and in certain cases by direct electrical stimulation of the cortex (Herbet et al., 2014). One remarkable example of drugs^1^ capable of inducing experiences with features common to dreaming is the family of serotonergic or “classical” psychedelics. These substances have been known and used in different parts of the world for millennia (Schultes and Hofmann, 1979; Metzner, 1998), but it is only after the synthesis (1938) and self-administration (1943) of lysergic acid diethylamide (LSD) by Swiss chemist Albert Hofmann that Western society became increasingly aware of their existence, leading to gradual but deep changes in psychiatry, culture and society (Hofmann, 1980). The action of serotonergic psychedelics is based on their high affinity for serotonin 5-HT2_A_ receptors (Glennon et al., 1984; Vollenweider et al., 1998; Kraehenmann et al., 2017; Preller et al., 2017) and is characterized by marked changes in consciousness that include simple and complex visual imagery, distortions in the sense of self and in the relationship between the body and the environment, disinhibited emotions, and alterations in cognition and thought processes (Schmid et al., 2015; Nichols, 2016).

While the experience elicited by serotonergic psychedelics has long been ascribed a dreamlike quality (Jacobs, 1978; Schultes and Hofmann, 1979; Fischman, 1983), only recently experiments in humans have provided evidence supporting a relationship between these drugs and REM sleep dreams (Carhart-Harris and Nutt, 2014; Carhart-Harris R. L. et al., 2014; Kraehenmann, 2017). This evidence comes mostly from neuroimaging experiments using LSD and psilocybin (the psychoactive compound behind the psychedelic effects of *Psilocybe* mushrooms) (Carhart-Harris et al., 2012, 2016; Tagliazucchi et al., 2014). Earlier studies demonstrated that LSD facilitates REM sleep in humans when administered during sleep or before sleep onset (Muzio et al., 1966; Torda, 1968; Green, 1969) and that N,N-dimethyltryptamine (DMT; an orally-inactive serotonergic psychedelic) induces spontaneous eye movements similar to those observed during REM sleep (Strassman, 2000).

In terms of rigorous analysis of the associated phenomenology (the first-person perspective of “what it feels like” to have an experience) evidence supporting a relationship between dreams and serotonergic psychedelics is scarcer. The recent work of Kraehenmann and colleagues established that LSD increases the “cognitive bizarreness” of mental imagery (Kraehenmann et al., 2017) (a characteristic quality of dream content; Hobson et al., 1987). Other studies have asked participants to explicitly self-assess the “dreamlike quality” of their psychedelic experience (Studerus et al., 2011; Carhart-Harris and Nutt, 2014; Carhart-Harris R. L. et al., 2014; Schmid et al., 2015; Dolder et al., 2016; Carhart-Harris et al., 2016). However, a quantitative and hypothesis-free comparison of first-person reports of psychedelic experiences and dreaming is currently lacking.

Other hallucinogen drugs^2^ acting through different pharmacological mechanisms can induce experiences that are also characteristic of REM sleep dream mentation. Dissociative psychedelics are chiefly synthetic anesthetic agents that disrupt the capacity for information transmission in the brain, even though such drugs can also be found in nature, e.g., muscimol, present in *Amanita muscaria* mushrooms. Examples include arylcyclohexamines ketamine and phencyclidine (PCP) (Morris and Wallach, 2014). When administered in sub-anesthetic doses, these drugs may lead to feelings of detachment from the body, self and environment, as well as perceptual distortions and hallucinations, depersonalization (feeling the self as unreal or lacking agency) and derealization (feeling the environment as unreal) (Hansen et al., 1988; Jansen, 1993; Malhotra et al., 1996; Pomarol-Clotet et al., 2006; Wilkins et al., 2011). Some of these experiences are frequent during normal dream episodes, especially the dissociation between the first-person point of view and the bodily self, while others are more characteristic of lucid dreams (e.g., derealization) (Hobson, 2009; Nir and Tononi, 2010; Thompson, 2014). Substances termed “deliriants” (Duncan and Gold, 1982) include the tropane alkaloids atropine, scopolamine, and hyoscyamine that are present in the flowering plants of the *Solanaceae* family (such as those in the *Brugmansia* and *Datura* genera) (Farnsworth, 1968; Schultes and Hofmann, 1979). The anticholinergic effect of these alkaloids leads to a state of delirium and confusion with hallucinations and complex visual imagery, in contrast to the relatively simple imagery experienced under the influence of serotonergic psychedelics (Safer and Allen, 1971; Osterholm and Camoriano, 1982; Bersani et al., 2013). Importantly, this imagery is frequently perceived as real and the users might not be aware that they are undergoing a drug-induced altered state of consciousness. This feature is common to dreams of low lucidity, during which the dreamer lacks the metacognitive capacity to identify the experience and its content as a dream (Kahan and LaBerge, 1994), but is absent in the experiences elicited by dissociative and serotonergic psychedelics (Nichols, 2016).

The phenomenological commonalities and divergences between dreaming and the effects of dissociative psychedelics and deliriants have received comparatively less attention than those of serotonergic psychedelics. The neurophysiological bases for these experiences and their relationship to those underlying REM sleep dream episodes also remain largely unexplored. In this work we seek to perform a comprehensive, large-scale analysis of subjective reports of the experiences elicited by a wide range of psychoactive substances including hallucinogens, as well as other drugs having less direct impact on the general quality of conscious experience (e.g., stimulants, sedatives, antipsychotics, antidepressants). Our main objective is to determine the semantic similarity between these reports and those of dreams, directly addressing the hypothesis that the experiences elicited by serotonergic psychedelics bear a high resemblance to dreaming (Carhart-Harris and Nutt, 2014; Carhart-Harris R. L. et al., 2014; Kraehenmann, 2017). More generally, we extend this hypothesis to encompass dissociative psychedelics and deliriants, and investigate whether the degree of similarity between the associated experiences and dreaming depends on the level of lucidity.

## Materials and methods

### Corpora selection

**Figure 1:**
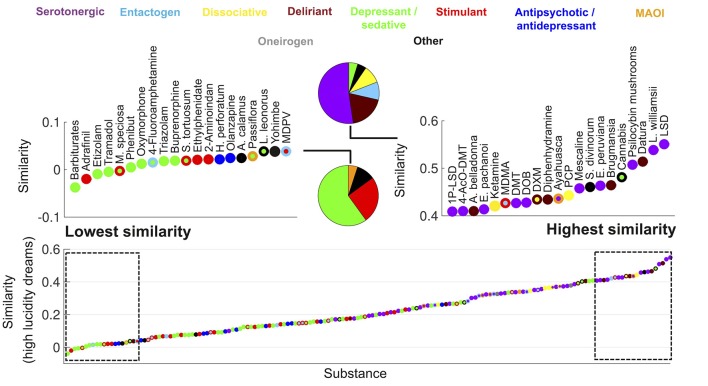
Ranking of psychoactive substances in the Erowid corpus in terms of the similarity of their subjective reports to those of high lucidity dreams (Dreamjournal corpus). The rectangles on the left/right zoom into the top 20 lowest/highest ranking substances and the pie charts indicate the proportion of each primary category. Substances are represented with circles that are color-coded based on their category (the color of the center/border corresponds to the primary/secondary category).

**Figure 2:**
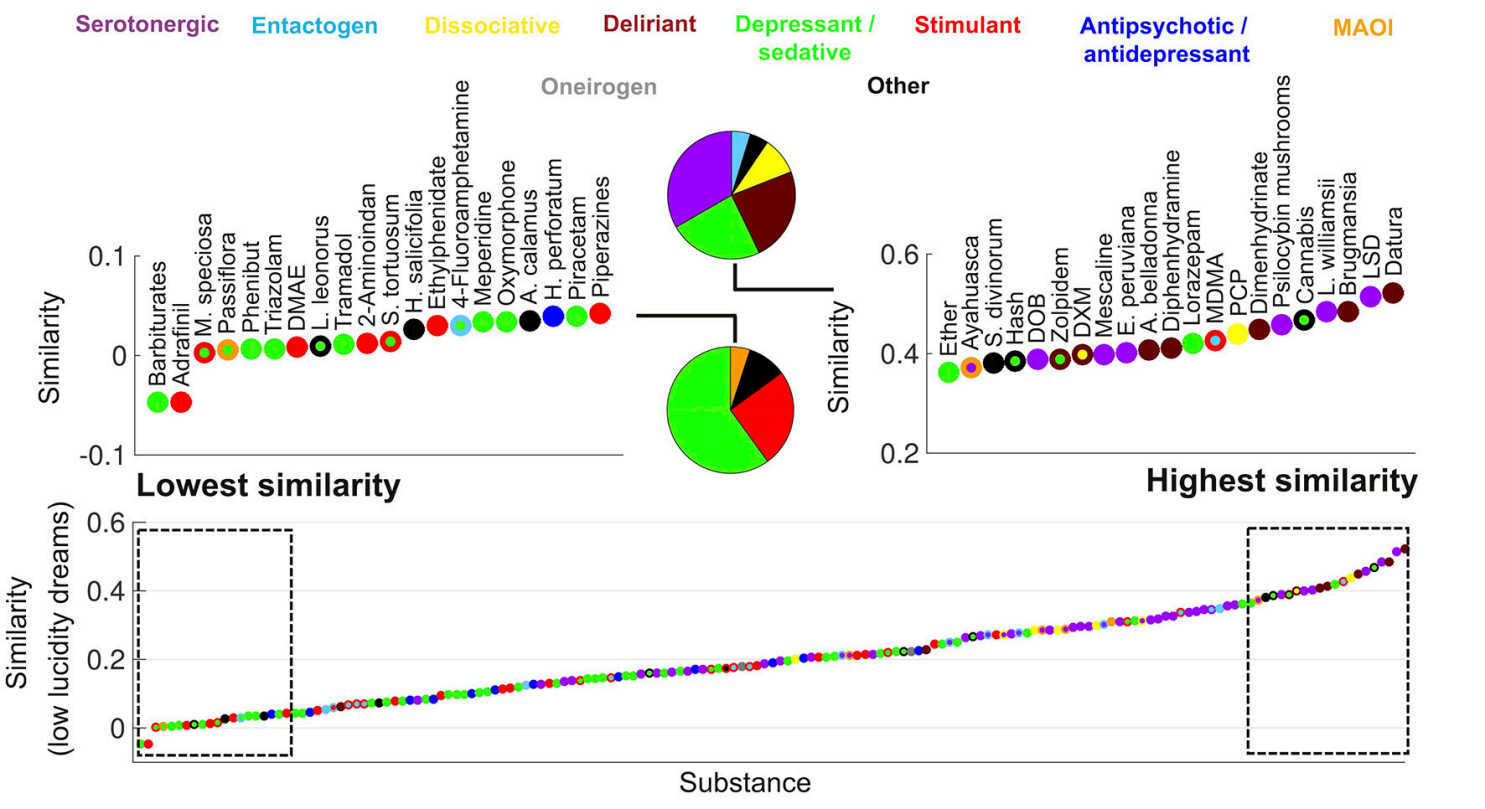
Ranking of psychoactive substances in the Erowid corpus in terms of the similarity of their subjective reports to those of low lucidity dreams (Dreamjournal corpus). The rectangles on the left/right zoom into the top 20 lowest/highest ranking substances and the pie charts indicate the proportion of each primary category. Substances are represented with circles that are color-coded based as in Figure 1.

Reports of psychoactive substance use were downloaded from the “experience vaults” in www.erowid.org and are here referred to as the “Erowid corpus.” The webpage www.erowid.org is a “member-supported organization providing access to reliable, non-judgmental information about psychoactive plants, chemicals, and related issues” containing, among other resources, a large number (>20,000) of reports associated with the use of different psychoactive substances. Our research relied upon Erowid's reviewed and edited collection of experience reports, and followed Erowid's terms of use that require researchers to coordinate with Erowid's research team in order to avoid misinterpretations of their data (https://erowid.org/general/about/about_copyrights.shtml). We discarded reports that resulted from the combination of different substances. When certain reports appeared under more than one category (e.g., under “phenylethylamine” and “2C-B”) we classified them in the most specific way unless such specificity dramatically reduced the number of reports associated with each individual substance (as in the case of psilocybin mushrooms, encompassing different species such as *Psilocybe mexicana* and *Psilocybe cyanescens*). We distinguished between reports of plants or fungi and their psychoactive compounds (e.g., between mescaline and cacti such as *Lophophora williamsii* and *Echinopsis pachanoi*). Finally, we only included substances that contained at least 10 reports. The names of all 165 psychoactive substances included in this study, together with the number of associated subjective reports, their similarity to dreams of low/high lucidity (see Figures 1, 2) and their primary and secondary categories are listed in Table 1.

**Table 1:** The 165 psychoactive substances included in the study, with the number of reports (N), their primary and secondary classification (the color-code is the same as in Figures 1, 2, and can be found in the bottom right of the table), and the ranking of the substances in terms of their similarity to high/low lucidity dreams.

	**N**	**Primary**	**Secondary**	**Rank (high/low)**	**Substance**	**N**	**Primary**	**Secondary**	**Rank (high/low)**
*Salvia divinorum*	1,267			8/19	Damiana	41			109/128
*Cannabis*	827			5/5	Valerian	41			123/124
MDMA	770			16/9	4-Ho-DiPT	40			77/98
LSD	718			1/2	Morphine	40			99/101
DXM	422			13/15	2C-D	40			80/100
Morning glory	334			38/34	Blue Lotus	40			113/131
2C-I	295			40/40	1P-LSD	40			21/27
Cocaine	289			89/71	DOB-DragonFLY	39			54/56
Amphetamines	250			71/50	6-APB	39			108/115
Datura	250			3/1	Ethylphenidate	38			154/153
Nutmeg	247			73/72	4-Acetoxy-DiPT	38			75/94
5-MeO-DMT	247			43/58	1,4-Butanediol	38			135/121
DMT	236			15/29	GBL	38			116/108
Metamphetamine	235			45/22	Heimia salicifolia	37			144/154
25I-NBOMe	233			46/43	Hash	37			25/18
Argyreia nervosa	224			51/42	DOB	36			14/17
Mitragyna speciosa	218			161/163	4-AcO-DET	36			83/113
2C-E	206			29/31	4-Fluoroamphetamine	35			158/152
5-MeO-DiPT	182			52/45	Acorus calamus	35			150/149
AMT	175			37/26	PCP	35			10/8
Tramadol	173			162/157	Cyclobenzaprine	33			115/116
Ketamine	164			17/25	DOI	32			72/82
2C-T-7	157			68/73	Yerba mate	32			125/117
2C-B	143			53/51	Iboga	32			33/53
Dimenhydrinate	143			12/7	Ibogaine	32			31/35
*Amanita muscaria*	142			35/41	AL-LAD	29			30/32
*Echinopsis pachanoi*	139			18/24	Hydromorphone	29			98/106
DPT	137			36/44	Oxymorphone	28			159/150
Heroine	133			74/61	A. peregrina	28			27/28
Caffeine	119			78/62	Trazadone	27			63/64
Nitrous oxide	118			34/49	Afrafinil	27			164/164
Synthetic cannabis	117			57/57	Opium	27			58/59
Zolpidem	114			26/16	MDAI	26			138/141
Oxycodone	111			117/102	Mirtazapine	25			122/127
Kava	104			95/107	Psychotria viridis	25			41/38
2C-T-2	101			69/78	Carisoprodol	25			81/75
Methoxetamine	95			39/46	Betel nut	24			93/76
Ayahuasca	93			11/20	5-HTP	24			119/122
GHB	88			106/97	Piracetam	24			124/147
Modafinil	86			134/132	Amitriptyline	23			96/79
MDPV	86			146/140	DOM	23			32/33
5-MeO-AMT'	84			88/89	Atropa belladonna	23			19/12
Gabapentin	76			136/125	*Lophophora williamsii*	23			2/4
Mephedrone	74			142/136	Sertraline	23			111/83
JWH-018	73			70/66	Ephedrine	22			112/95
Mimosahuasca	72			50/48	Zopiclone	22			86/81
Tobacco	70			97/77	Piperazines	21			139/146
*Brugmansia*	69			6/3	4-Acetoxy-MiPT	21			55/52
Opium (poppies)				132/133	Coffee	20			76/54
*Calea zacatechichi*	68			28/65	TFMPP	20			110/118
Methylphenidate	68			107/85	DMAE	20			137/159
Hydrocodone	64			103/90	Butylone	20			102/86
MDA	63			44/30	*Banisteriopsis caapi*	19			42/39
Methylone	62			94/104	Phenibut	19			160/161
Paroxetine	61			128/114	TMA-2	19			49/55
Venlafaxine	60			145/130	Lorazepam	19			22/10
Methadone	60			133/120	Olanzapine	18			151/143
2C-C	60			82/96	Diazepam	18			105/103
Fentanyl	59			120/123	Passion flower	17			149/145
Alcohol	58			84/68	Benzylpiperazine	17			114/112
Alcohol (hard)	57			59/37	Etizolam	17			163/144
Echinopsis peruviana	56			7/13	Mescaline	16			9/14
Alprazolam	55			66/36	Alcohol (beer)	16			100/91
4-AcO-DMT	55			20/23	Atomoxetine	15			141/142
Melatonin	54			60/105	*Hypericum perforatum*	15			152/148
Bupropion	54			104/93	MBDB	15			126/137
Codeine	54			118/111	2-Aminoindan	14			152/156
Cannabinoid agonists	53			91/99	Scopolamine	14			62/63
Buprenorphine	52			156/145	3-MeO-PCP	13			64/80
Crack	48			85/69	Propylhexedrine	13			129/126
Sceletium tortuosum	48			155/155	2C-T-4	12			92/110
A. colubrina	48			79/92	Triazolam	12			157/160
Dipt	45			67/84	Barbiturates	12			165/165
2C-P	45			56/60	IAP	12			101/88
Catnip	45			130/134	Meperidine	12			140/151
Ether	45			23/21	Ethylone	12			143/138
DOC	45			47/47	2C-T-21	12			121/129
Yohimbe	45			137/149	*Silene undulata*	11			48/87
Leonotis leonurus	44			148/158	**Serotonergic**			**Dissociative**	
5-MeO-DALT	44			87/109	**Entactogen**			**Depressant/sedative**	
Quetiapine	44			127/119	**Stimulant**			**Deliriant**	
5-MeO-MIPT	41			65/74	**MAOI**			**Antipsychotic/antidepressant**	
4-HO-MiPT	41			61/70	**Oneirogen**			**Other**	

In determining the categories we adopted a hybrid criterion based on pharmacological action and the subjective effects induced by the substances. Serotonergic or “classical” psychedelics (5-HT_2A_ agonists) were grouped based on their mechanism of action (even though their subjective effects are generally difficult to discriminate; Wolbach et al., 1962). The category of dissociative psychedelics comprised primarily NMDA antagonists such as ketamine and PCP, but also included substances with other mechanisms of action (e.g., *Amanita muscaria* mushrooms). The same applies to the case of deliriants, which in most cases were *Solanaceae* plants rich in tropane alkaloids. Entactogen drugs were categorized primarily by their subjective effects [a representative drug in this category is “ecstasy” or 3,4-methylenedioxymethamphetamine (MDMA)]. Stimulants included dopaminergic drugs such as cocaine, amphetamines and modafinil, as well as others of different pharmacological profile. Similarly, depressants/sedatives were defined by their effect on the central nervous system and included substances such as benzodiazepines as well as different natural and synthetic opioid analgesics. Prescription antidepressants and antipsychotics (also including plants of antidepressant effect, such as *Hypericum perforatum* or St. John's wort) were grouped together into one category. While only two plants in the Erowid corpus are consumed primarily for their oneirogen effect (*Calea zacatechichi* and *Silene undulata*) we created a category that includes them, given their relevance for the present study. Certain substances had a large number of subjective reports but their relatively unique mechanism of action did not justify the creation of a new category, such substances were classified as “other.” Examples include plants of the *Cannabis* genus and cannabinoid receptor agonists, and *Salvia divinorum*. Finally, all substances were given a primary and a secondary category (even though in many cases those were identical) based on different facets of their subjective effects (e.g., MDMA was classified primarily as an entactogen and secondarily as an stimulant) or the fact that their mechanism of action depends on the combination of substances of different categories [e.g., the psychedelic effects of ayahuasca result from the combination of plants rich in the orally-inactive serotonergic psychedelic DMT with beta carbolines acting as monoaminooxidase inhibitors (MAOI) present in the liana *Banisteriopsis caapi*].

Dream reports were obtained from www.dreamjournal.net, a free service with over 15,000 users and over 200,000 dream reports. Besides the dream narratives themselves, some reports include additional information such as the level of lucidity, cohesion (both rated from one to five points) and whether the dreamer had the intent of achieving lucidity. The level of lucidity was used to separate dreams of low (1 point) and high (5 points) lucidity. A total of 2,914,498/716,189 words comprising reports of low/high lucidity dreams were obtained for the present analysis and comprise the “Dreamjournal corpus.”

While both www.erowid.org and www.dreamjournal.net are curated to avoid the posting of unrelated or poor quality content, it is impossible for the curators to assess the validity of the circumstances associated with the reports (e.g., the identity of the consumed substance or its dosage, or whether dreamers achieved lucidity or not). This point is further discussed below (see “Methodological Considerations and Limitations” in the Discussion section).

### Corpora preprocessing

The preprocessing of the text corpora was performed using the Natural Language Toolkit (NLTK, http://www.nltk.org/) in Python 3.4.6 (Bird, 2006). Both corpora of subjective reports were first separated into individual words after discarding all punctuation marks (word repetitions were allowed). Each word was lemmatized using NLTK (i.e., converted to the root from which it is inflected). All words were converted to lowercase and lemmatized words containing less than three characters were discarded.

Since texts from the Erowid corpus are likely to be influenced by the nature of the substance being reported, we compiled a list of words including substance names, different slang variations, and words relating to the possible routes of administration. A total of 12,465 words fulfilling these criteria were manually selected and the lemmatized versions of these words were discarded from the Erowid corpus. The rationale between this “censoring” of the corpus was to retain words relating to the experienced effects but not to the surrounding circumstances prior to the use of the substances.

### Latent semantic analysis

The basis for the present analysis is comparing the profile of word frequencies between reports of each substance from the Erowid corpus and dream reports of high/low lucidity from the Dreamjournal corpus. As a first approximation, we estimated that if the word occurrence frequencies of two texts are highly correlated, the topics being described in those texts must be related. Note that this analysis is not based on the frequency of individual words, but on the relationship between all frequencies in each pair of texts.

For a large vocabulary of terms the occurrence frequencies are likely to be sparse, i.e., most terms will not appear in either text and therefore their frequency will be zero. To avoid this situation we used Latent Semantic Analysis (LSA) (Landauer, 2006), a natural language processing technique based on the hypothesis that words with similar meaning appear with similar frequency in texts (Sahlgren, 2008). Before applying LSA we computed the frequency of the different words using the term frequency–inverse document frequency (tf-idf) transform, as implemented in scikit-learn (www.scikit-learn.org; Leskovec et al., 2014). The tf-idf transform computes a matrix in which rows are unique words in the corpus and columns represent “documents” (in this case, each document is either a substance from the Erowid corpus or the collection of high/low lucidity dreams from the Dreamjournal corpus). The product of the term frequency and the inverse document frequency determines the entries of this matrix. The term frequency is defined as the count of times each term appears in each document. The inverse document frequency is defined as the logarithmically scaled inverse fraction of the documents containing the term. To eliminate very frequent/rare terms from the corpus, only those terms appearing in more/less than 5%/95% of the documents were retained.

To apply LSA, the word-by-document matrix obtained using the tf-idf transform was decomposed into the product of three matrices using Singular Value Decomposition (SDV; Klema and Laub, 1980). Of the three resulting matrices (U, S, V), S contains in its diagonal the matrix of singular values ordered by size, and U and V are real unitary matrices (their size is determined by the number of words and documents, respectively). To reduce the number of linearly independent rows (terms) while preserving the similarity structure among columns (documents), only the first D largest singular values were retained and all others set to zero, resulting in the reduced matrix of singular values S^*^. We retained the D = 20 largest singular values (a comparison of the results using different choices of D is shown in **Figure 8**). Computing the product of U, S^*^, V yields a low-rank approximation of the word-by-document frequency matrix, which mitigates the problem of sparseness and provides the similarity between documents based on context-sensitive term occurrence. For instance, even though the sentences “the garden was full of flowering roses” and “a vase with daisies sits on the table” share no words in common, they will be identified as similar as they both relate to the term “flower.” Previous work has established the adequacy of LSA to classify the subjective effects of different psychoactive substances (Bedi et al., 2014).

Finally, after obtaining the rank-reduced version of the term frequency matrix we computed the semantic similarity between the subjective reports of each substance in the Erowid corpus and the reports of high/low lucidity dreams by computing the Pearson linear correlation coefficient between the associated columns of the matrix.

## Results

In Figure 1 we show all 165 substances from the Erowid corpus ranked according to the similarity of their associated reports to those of high lucidity dreams. Each substance is represented as a colored point, the center of each point is color-coded based on the primary category of the substance and the border is color-coded based on its secondary category. For instance, ayahuasca is represented with a purple center and an orange border, indicating it is a combination of a MAOI that enables the psychedelic effects of DMT. The left (“Lowest similarity”) and right (“Highest similarity”) panels zoom into the top and bottom 20 substances according to the similarity of their subjective effects to high lucidity dreaming. The pie charts indicate the proportion of substances of each primary category within the top and bottom 20 drugs. The highest-ranking substance was LSD, followed by *Lophophora williamsii* (peyote, a cactus containing the serotonergic psychedelic mescaline) and then plants of the *Datura* genus (containing deliriant tropane alkaloids). The highest-ranking dissociative psychedelic was PCP. Among the top 20 substances the only ones that were not classified as hallucinogens were plants of the *Cannabis* genus whose main psychoactive effects are mediated by tetrahydrocannabinol (a partial agonist of the cannabinoid receptors CB_1_ and CB_2_; Kumar et al., 2001), *Salvia divinorum* (a psychoactive plant capable of producing intense alterations in consciousness mediated by salvinorin-A, a kappa opioid receptor agonist; Roth et al., 2002) and MDMA (a substituted amphetamine with entactogen and stimulant effects produced by facilitation of the presynaptic release of serotonin, norepinephrine and dopamine; Nichols, 1986). As shown in the pie chart, hallucinogens accounted for almost 80% of the top 20 substances. Conversely, the bottom 20 substances included neither dissociative/serotonergic psychedelics nor deliriants, and consisted mostly of depressant/sedatives such as babiturates, benzodiazepines/benzodiazepine analogs (etizolam, triazolam) and opioids (tramadol, oxymorphone), stimulants (adrafinil, ethylphenidate, 2-Aminoindane) and antidepressant/antipsychotics (*Hypericum perforatum* and olanzapine).

Figure 2 shows the same information as Figure 1 but for the similarity to low lucidity dreams. The top ranked substances included a larger proportion of deliriants. Plants of the *Datura* and *Brugmansia* genera ranked first and third, respectively. Dimenhydrinate (a medication used to treat motion sickness that is recreationally abused due to its deliriant properties at high doses; Halpert et al., 2002) was among the top ranked substances, while it was absent for the similarity to high lucidity dreams (Figure 1).

It is interesting to note that melatonin, *Silene undulata* and *Calea zacatechichi* were the three substances with the highest difference in their similarity between high and low lucidity dreaming. The latter two are plants traditionally employed as oneirogens and purported to increase dream lucidity (Schultes and Hofmann, 1979), whereas melatonin is a hormone endogenous to the human body that has been explored as a lucidity-enhancing agent (La Berge, 2003). Conversely, the three drugs that resulted in less “lucid” experiences (according to the comparison of their associated reports with those of high vs. low lucidity dreams) were alprazolam, sertraline, and clonazepam. The first and third of these drugs are benzodiazepines, while sertraline is an antidepressant of the selective serotonin reuptake inhibitor class.

**Figure 3:**
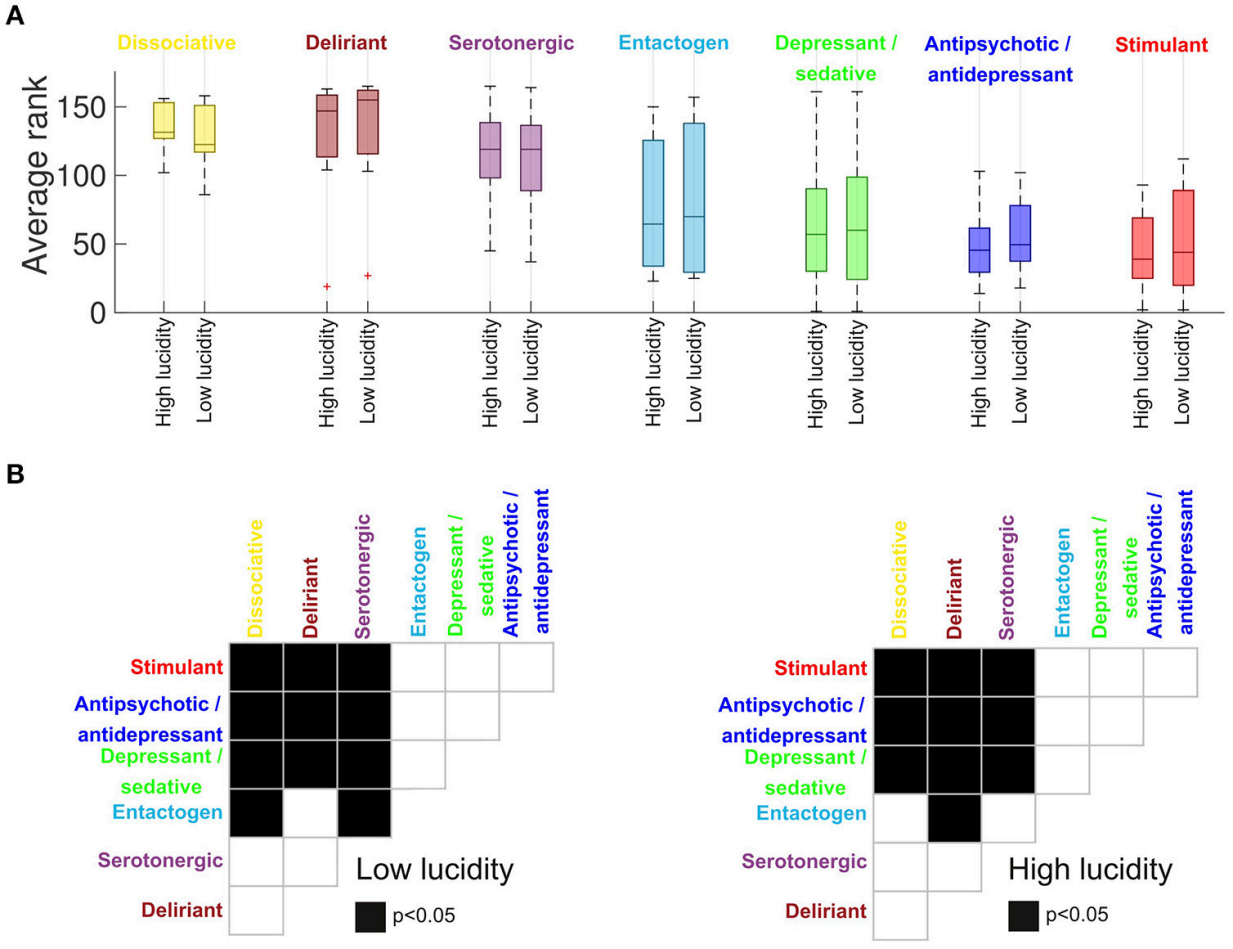
**(A)** Average substance rank (per category) in terms of the similarity of the reported effects to high/low lucidity dream reports (median ± 25th and 75th percentiles, “+” represents outliers). Higher numbers correspond to higher similarity (the highest possible rank is 165). The categories “oneirogen,” “MAOI,” and “other” were excluded from this figure due to their small sample size (*n* ≤ 6). The drug category presented a significant effect on the similarity with dream reports (*F* = 31.34, *p* < 0.0001, multi-factor ANOVA), while the interaction between dream lucidity and similarity with dream reports was non-significant (*F* = 0.02, *p* = 0.88). **(B)** *Post-hoc* Wilcoxon signed-rank tests for the difference in the similarity to dreams for each pair of drug categories. Black squares represent significant differences (*p* < 0.05) for the pair of drug categories in the corresponding rows and columns.

Figure 3A shows the average rank of all substances divided by category and by the similarity of their reports to those of high/low lucidity dreams (the categories corresponding to MAOI, oneirogens and others were excluded due to their small number of substances). The top three categories were dissociative psychedelics, serotonergic psychedelics and deliriants, followed by entactogens, depressant/sedatives, antipsychotic/antidepressants and stimulants. A multi-factor analysis of variance (ANOVA) was conducted with the ranking of the drugs as dependent variable and two grouping variables (drug category and dream lucidity). A significant effect of drug category on the similarity to dreams was observed (*F* = 31.34, *p* < 0.0001), while the interaction between dream lucidity and similarity with dream reports was non-significant (*F* = 0.02, *p* = 0.88). Afterwards we conducted *post-hoc* Wilcoxon signed-rank tests for the difference in the similarity to dreams for each pair of drug categories (Figure 3B). We observed significant differences between all hallucinogen drugs and depressants/sedatives, antipsychotic/antidepressants and stimulants. No significant differences were found among hallucinogens or among the group of depressants/sedatives, antipsychotic/antidepressants and stimulants. Entactogen reports were significantly less similar to dream reports for some hallucinogen drugs, which depended on the level of dream lucidity.

**Figure 4:**
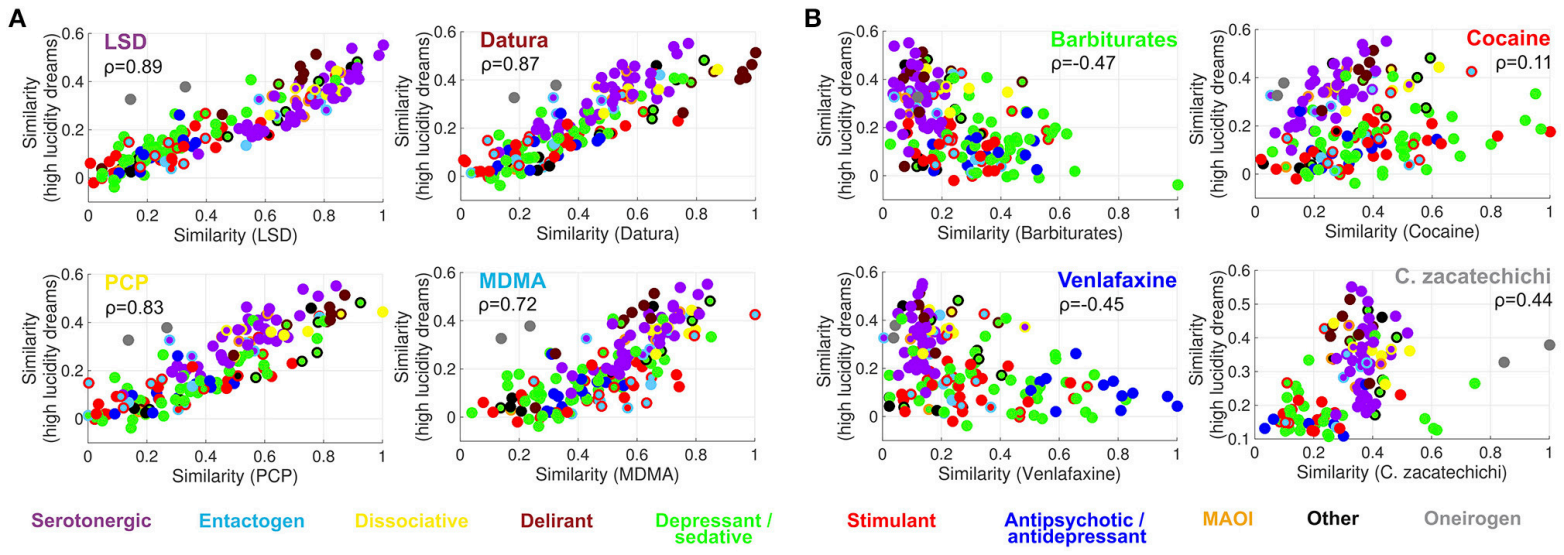
Prediction of the similarity between the subjective report of substances and dreaming experiences of high lucidity, based on the similarity to LSD, plants of the *Datura* genus, PCP and MDMA **(A)** and to reports of barbiturates, cocaine, venlafaxine and *Calea zacatechichi* **(B)**. Substances are represented with circles that are color-coded as in Figure 1. The inset shows the value of the Spearman's rank correlation coefficient (ρ).

**Figure 5:**
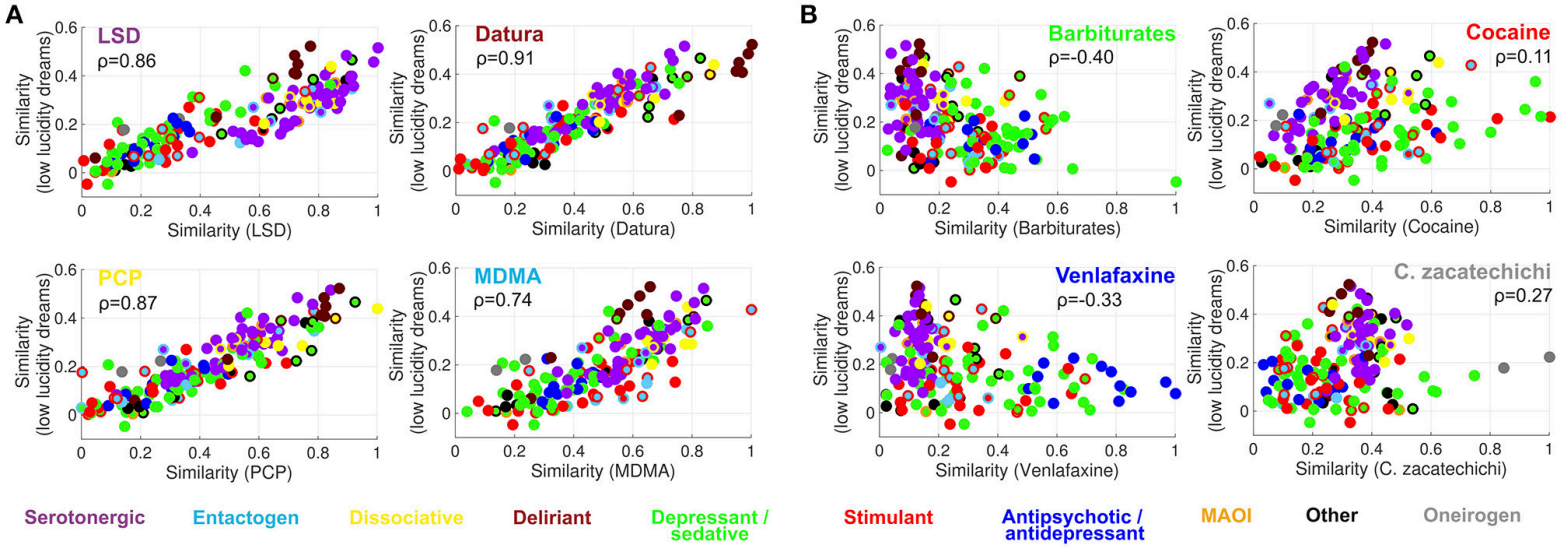
Prediction of the similarity between the subjective report of substances and dreaming experiences of low lucidity, based on the similarity to LSD, plants of the *Datura* genus, PCP and MDMA **(A)** and to reports of barbiturates, cocaine, venlafaxine and *Calea zacatechichi* **(B)**. Substances are represented with circles that are color-coded as in Figure 1. The inset shows the value of the Spearman's rank correlation coefficient (ρ).

Figure 4A shows that it is possible to predict the semantic similarity between reports of different substances based on their semantic similarity to dreams of high lucidity. Four high-ranking substances belonging to different categories (LSD, plants of the *Datura* genus, PCP and MDMA) were selected. Each point in the scatter plots represents a substance in the Erowid corpus (color-coded as in Figures 1, 2) with its X axis coordinates indicating the similarity of its subjective reports to those of each of the four selected substances and its Y axis coordinates to reports of high lucidity dreams. The correlation coefficient between both similarity indices (Spearman correlation,ρ) is shown as an inset [LSD (ρ = 0.89), *Datura* genus (ρ = 0.87), PCP (ρ = 0.83), MDMA (ρ = 0.72)]. Figure 4B shows the same information for four substances that rank lower in terms of their similarity to high lucidity dream reports: barbiturates, cocaine, venlafaxine and *Calea zacatechichi*. In contrast to the scatter plots shown in Figure 4A it is clear that a lower correlation exists between both similarity indices [Barbiturates (ρ = −0.47), Cocaine (ρ = 0.11), Venlafaxine (ρ = −0.45), *Calea zacatechichi* (ρ = 0.44)]. Figure 5 shows the same information as Figure 4 but for the similarity to low lucidity dreams [LSD (ρ = 0.86), *Datura* genus (ρ = 0.91), PCP (ρ = 0.87), MDMA (ρ = 0.74), Barbiturates (ρ = −0.40), Cocaine (ρ = 0.11), Venlafaxine (ρ = −0.33), *Calea zacatechichi* (ρ = 0.27)].

**Figure 6:**
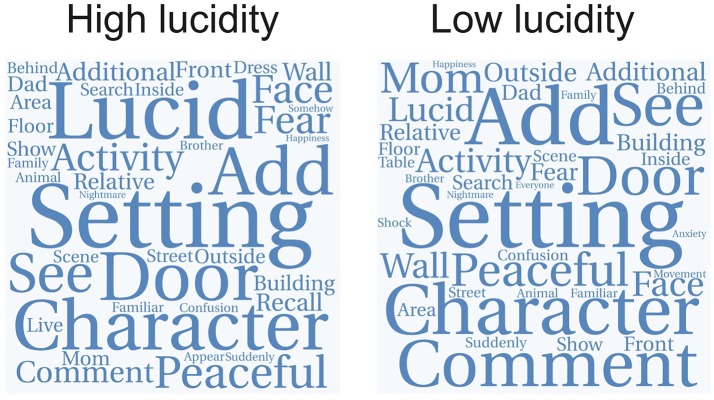
Word clouds for the top 40 most frequent terms in dream reports of high **(left)** and low **(right)** lucidity.

After establishing that subjective reports of hallucinogens present the highest semantic similarity to dream reports, we investigated the most frequent terms within dreams of high and low lucidity, and for the selection of substances presented in Figures 4A, 5A. Figure 6 shows a word cloud representation of the frequency of the most 40 common terms in the vocabulary of high (left panel) and low (right panel) lucidity dream reports (all word clouds were generated using www.wordart.com). The most common terms in both cases related to the setting/location (the term “setting” itself, as well as others such as “door,” “street,” “outside,” “wall,” “behind”), emotions (“fear,” “peaceful,” “happiness,” “confusion,” “anxiety”), relatives (“mom,” “dad,” “brother”) and perception (“see,” “face,” “movement”). The main difference between both sets of reports related to the frequency of the term “lucid” itself, which possibly reflected the fact that Dreamjournal users explicitly commented on their intents of achieving lucid dreaming.

**Figure 7:**
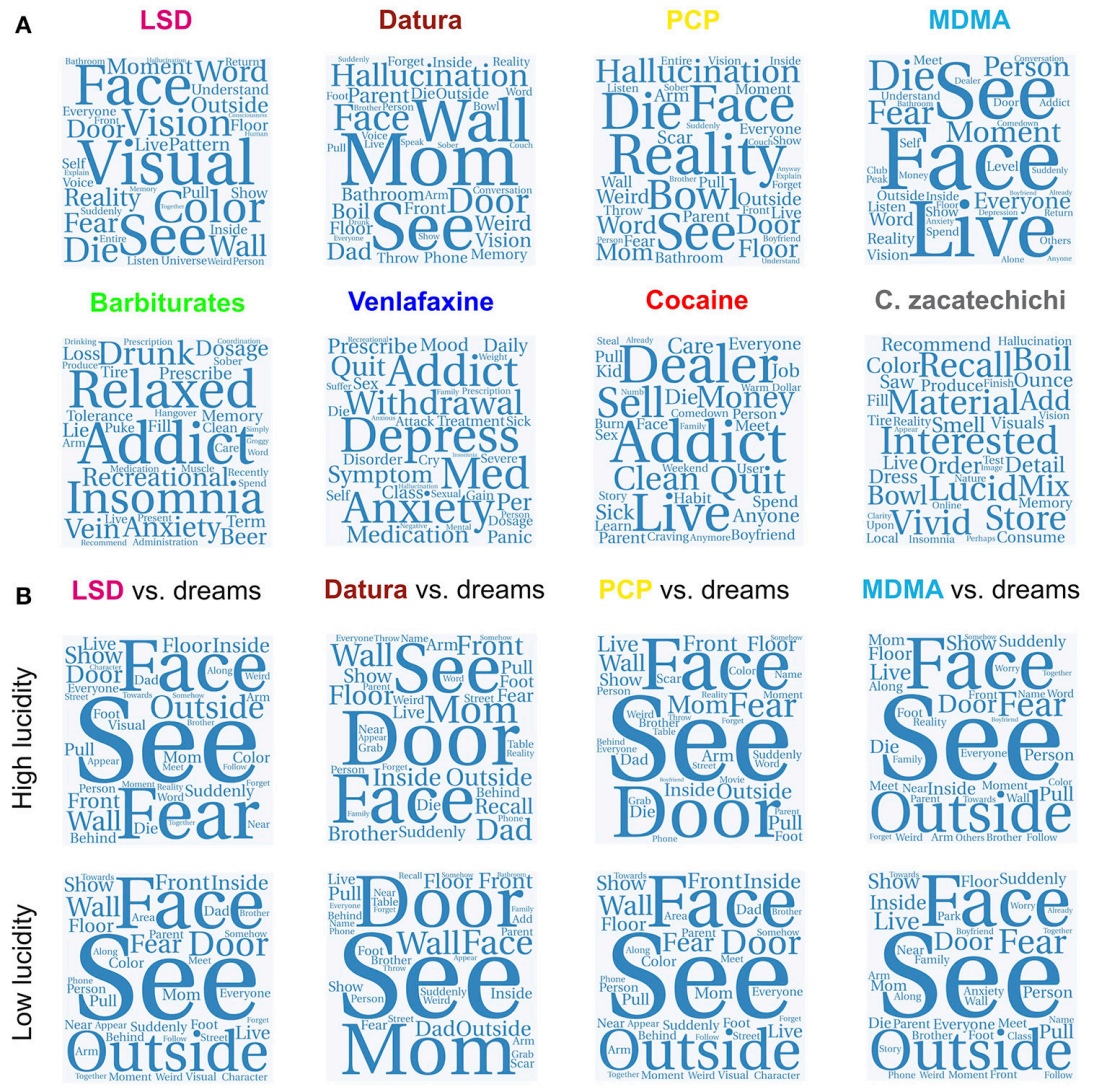
Word clouds for the top 40 most frequent terms in the reports of four substances with high similarity to dream reports (LSD, plants of the Datura genus, PCP and MDMA) and in the reports of four substances with lower similarity to dream reports (barbiturates, venlafaxine, cocaine, and *Calea zacatechichi*) **(A)**. **(B)** shows word clouds based on the average ranking of each term in the reports of the four psychoactive substances of **(A)** and in the reports of high/low lucidity dreams.

We produced similar word clouds for the reports of substances with high (LSD, plants of the *Datura* genus, PCP and MDMA) and low (barbiturates, cocaine, venlafaxine and *Calea zacatechichi*) similarity to dream reports (Figure 7A). Consistent with the effects of these substances, high-frequency terms in reports of hallucinogens related to distortions in visual perception, emotions, and the setting of the experiences. The term “hallucination” did not appear among the most frequent for LSD reports, consistent with the fact that serotonergic psychedelics are characterized by relatively simple visual imagery. MDMA reports also included terms that relate to the typical setting of entactogen use (e.g., “club,” “boyfriend,” “alone,” “conversation,” “person”). Substances with reports of low similarity to dreaming included terms that did not relate to perception/setting/consciousness/emotion but depended on the substance, its effect and the general circumstances of its most common recreational use. For instance, barbiturate reports included frequent terms related to their effects and their intended medical use (“relaxed,” “insomnia,” “prescribe”), as well as to their addictive potential (“tolerance,” “sober,” clean”). Similar terms appeared for cocaine, another substance with addictive potential (“addict,” “quit,” “craving”). Frequent terms related to *Calea zacatechichi* vaguely indicated its use as an oneirogen (“lucid,” “vivid,” “visuals”), but seemed to relate mostly to the preparation of the substance and recommendations for other Erowid readers (“recommend,” “bowl,” “boil,” “ounce,” “material,” “mix,” “store,” “fill,” “add”).

We computed the rank of each term in the vocabulary (in terms of its frequency in the rank-reduced tf-idf matrix) for reports of LSD, plants of the *Datura* genus, PCP and MDMA, as well as for reports of high/low lucidity dreaming. We averaged both ranks and produced a word cloud in which term size is weighted by the average rank, i.e., terms that rank high both in substance and dream reports appear with the largest sizes. These word clouds are shown in Figure 7B. The most prevalent terms related to facets of the experience that were both relevant for hallucinogens and dreams. These included terms associated with perception (“see,” “visual,” “face,” “reality,” “color”), emotion (“fear”), setting (“outside,” “inside,” “street,” “front,” “behind”) and relatives (“mom,” “dad,” “brother,” “parent,” “family”).

**Figure 8:**
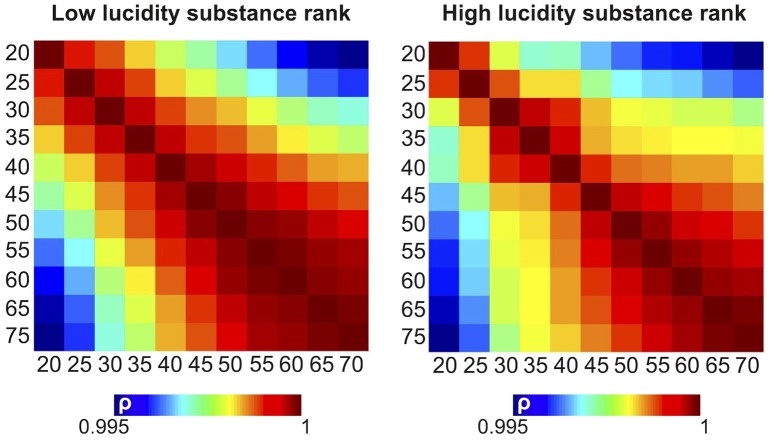
Pairwise correlation matrices between the rank of the substances in terms of their similarity to dreams of low (left) and high (lucidity), computed after retaining different numbers of singular values.

The results shown in the previous figures are based on retaining the components associated with the 20 largest singular values (*D* = 20) after performing SVD. To investigate the parametric dependence of the results upon the number of retained singular values, we repeated all analyses using *D* = 20, 25, 30, 35, 40, 45, 50, 55, 60, 65, and 70. We then computed the Spearman correlation coefficient between the substance rankings obtained for each pair of number of singular values. The resulting correlation matrices are shown in Figure 8 for dreams of low lucidity (left) and high lucidity (right). While the highest correlation coefficients appeared close to the diagonal (indicating that the ranking of the substances changed continuously with the relative difference of the number of retained singular values), all correlation coefficients were larger than 0.995, meaning that the substance rankings were highly stable for the different choices of this parameter.

## Discussion

We have applied techniques from natural language processing to a large corpus of subjective reports to investigate the hypothesis that, among a wide range of psychoactive substances, hallucinogens lead to experiences that are most similar to those reported during dreaming. Anecdotal and historical evidence -supported by neurophysiological observations- led to the formulation of the aforementioned hypothesis by different authors (Jacobs, 1978; Schultes and Hofmann, 1979; Fischman, 1983; Carhart-Harris and Nutt, 2014; Carhart-Harris R. L. et al., 2014; Kraehenmann, 2017). We support it for the first time with phenomenological evidence based on subjects freely reporting the nature of their experiences. In the following we discuss dreaming and drug-induced alterations in consciousness for three separate domains: changes in sensory perception, self-awareness, and metacognitive function. We also discuss the consistency of our results with our current phenomenological and neurophysiological understanding of dreaming, and their contribution toward a better characterization of dreaming and drug-induced altered states of consciousness.

### Changes in sensory perception

Dreams and certain psychoactive substances can bring about changes in perception, predominantly visual but also auditory, tactile and proprioceptive (Hobson, 2009; Nir and Tononi, 2010; Thompson, 2014; Nichols, 2016). Dreams occurring during REM sleep frequently involve complex and vivid imagery that may not be recognized as a departure from ordinary conscious wakefulness. Dreams are highly visual experiences, full of rich imagery comprising colored and moving objects identical or similar to those frequently perceived during wakefulness (Hobson, 1988). While it has been argued that visual imagery during REM sleep can be characterized as a hallucination (i.e., as bottom-up perception Hobson, 1992a), Thompson, Nir and Tononi have eloquently defended the position that its phenomenology is closer to that of spontaneous imagination (i.e., top-down perceptual imagery Nir and Tononi, 2010; Thompson, 2014).

In contrast, serotonergic psychedelics tend to produce subtler perceptual modifications that cannot be easily classified in either category and that are frequently described as “simple” visual imagery (Nichols, 2004, 2016) [however, some exceptional serotonergic psychedelics such as N,N-diisopropyltryptamine (DiPT) primarily induce auditory distortions; Shulgin and Carter, 1979]. The nature of simple visual imagery elicited by serotonergic psychedelics is consistent with their effect at cortical areas early in the visual hierarchy (Ermentrout and Cowan, 1979; Bressloff et al., 2002; de Araujo et al., 2012; Carhart-Harris et al., 2016; Kometer and Vollenweider, 2016; Roseman et al., 2016). In addition, changes in higher visual areas have been reported for the acute effects of serotonergic psychedelics. For instance, psilocybin increased glucose metabolism in the temporal lobe, which might indicate altered processing of visual information along the ventral stream (Vollenweider et al., 1997). Psilocybin also modified relatively late evoked potentials associated with simple and complex visual imagery (Kometer et al., 2011, 2013). We can speculate that the complexity of visual imagery depends on both the dosage and the nature of the drug. For instance, relatively high doses may increase the dreamlike character of visual imagery, and certain serotonergic psychedelics (such as DMT) are prone to produce stronger and interactive complex imagery, especially with eyes closed (Strassman et al., 1994; Strassman, 2000; Shanon, 2002; Luke, 2011). Since it is difficult to obtain reliable information on dosage from the Erowid corpus (see the “Methodological Considerations and Limitations” section), the relationship between psychedelic-induced complex visual imagery and dream mentation should be investigated in the future using more controlled experimental paradigms.

The most frequent visual modifications induced by serotonergic psychedelics are elementary in nature and include color and pattern recognition enhancement (Hartman and Hollister, 1963; Oster, 1966), the presence of trails behind moving objects (Dubois and VanRullen, 2011), drifting of the visual field, and imagery that is predominantly geometric in nature (Klüver, 1942; Siegel and Jarvik, 1975; Kometer and Vollenweider, 2016; Roseman et al., 2016). This last feature of perceptual distortions has been explained by the form of the retino–cortical map and the architecture of the human primary visual cortex (Ermentrout and Cowan, 1979; Bressloff et al., 2002; Kometer and Vollenweider, 2016). These observations suggest that visual distortions elicited by typical recreational doses of serotonergic psychedelics and REM sleep dreams might differ in terms of their complexity and similarity to the visual content typical of conscious wakefulness. In spite of these differences, a close relationship between visual imagery during sleep and the serotonin system is suggested by the observation that serotonergic antidepressants alleviate the presence of complex visual hallucinations in patients with narcolepsy, a neurological disorder leading to the abnormal occurrence of REM sleep episodes (Manford and Andermann, 1998).

The differences in the nature of visual imagery elicited by serotonergic psychedelics and dreaming are manifest in the word clouds shown in Figure 7A. In the example of LSD, the most frequent terms are related to visual perception and to the act of seeing/perceiving itself (“see,” “visual,” “vision”), to simple visual imagery (“color,” “pattern”) and to more complex concepts that could either be the content of complex visual imagery or part of the setting (“face,” “door”). This is consistent with experimental studies showing that LSD produced more elementary compared to complex hallucinations (Carhart-Harris et al., 2016; Kometer and Vollenweider, 2016; Liechti et al., 2017). High-frequency terms related to simple visual imagery were absent in the reports from plants of the *Datura* genus, PCP and MDMA. When ranking terms based on their joint frequency in LSD and dream reports (Figure 7B) terms related to simple visual imagery lost prominence.

Dissociative psychedelics are not known to produce strong perceptual modifications at low doses; however, higher dosage can lead to a state of perceptual dissociation from the environment that is characterized by intense and complex visual hallucinations (Muetzelfeldt et al., 2008). In the case of ketamine, such state is colloquially referred to as “k-hole” and also results in alterations in self-awareness and the relationship between the body boundaries and the environment (to be discussed below). Deliriant plants rich in tropane alkaloids such as those of the *Datura* and *Brugmansia* genera produce complex visual hallucinations that are convincing to the user. In certain cases, the user might even interact with such hallucinations and completely lose awareness of undergoing a sensory disconnection with the environment (Safer and Allen, 1971; Osterholm and Camoriano, 1982; Bersani et al., 2013; Schmid et al., 2015). Thus, these substances might generate visual content highly similar to that experienced during dreaming. It must be noted, however, that the incapacity to identify the hallucinatory character of visual content is not a requisite for experiencing vivid and complex imagery. For instance, such imagery is experienced during dreams of high lucidity, even though dreamers are aware of the nature of their experience. Pharmacologically, realistic eyes-closed visual imagery (referred to as “brain movies” by Shulgin; Shulgin and Shulgin, 1995) can be induced by substituted amphetamines of entactogen effect such as 3-methoxy-4,5-methylenedioxyamphetamine (MMDA), even though the user easily identifies such content as artificial.

Psychoactive substances belonging to the other categories included in this study are not primarily characterized as hallucinogenic and do not routinely induce perceptual modifications. Certain stimulants such as metamphetamine can occasionally induce confusional states that include multimodal hallucinations, but these states are not among the sought-after effects of the drugs and tend to be exceptions (McKetin et al., 2006). Other substances such as alcohol, barbiturates and benzodiazepines are known for their capacity to induce withdrawal syndromes including features common to the experiences elicited by deliriant alkaloids, such as confusion, delusions and convincing multimodal hallucinations (Sellers, 1988; Schuckit, 2014). Again, these states do not represent normal recreational use and are not likely to inflate the similarity between the subjective reports of these substances and those of dreams. Thus, our knowledge of how substances of different categories impact on sensory perception is consistent with the semantic similarity between subjective reports of hallucinogens and dreams, with the caveat that deliriants could generate visual hallucinations closer to those experienced during dreams than those elicited by serotonergic psychedelics.

### Serotonergic psychedelics, dreams, and cortical inhibition

While the neurophysiological underpinnings of drug-induced simple and complex visual imagery remain to be completely understood, recent neuroimaging experiments suggest that they relate to enhanced coupling between primary visual areas and higher cortical regions and to increased thalamocortical functional connectivity (Carhart-Harris et al., 2016; Müller et al., 2017). Such changes are likely mediated by the activation of serotonin 5-HT_2A_ receptors, as activation of 5-HT_2A_ receptors has been identified as the key mechanism of action of serotonergic hallucinogens (Glennon et al., 1984; Vollenweider et al., 1998; Rickli et al., 2016; Kraehenmann et al., 2017; Preller et al., 2017). These receptors are highly expressed in posterior brain regions linked to visual information processing (Saulin et al., 2012), causing an increase in the excitability of layer V cortical pyramidal neurons projecting to inhibitory interneurons (Andrade and Weber, 2010; Bastos et al., 2012). The highly visual nature of psychedelic experiences and REM sleep dreams is supported by increased occipital metabolism and cerebral blood flow during both states (Braun et al., 1997, 1998; Carhart-Harris et al., 2016)—but a recent report showed decreased occipital blood flow under psilocybin, which could be related to dose, effects specific to this drug, or to differences in the data acquisition method (Lewis et al., 2017). These variables aside, solid experimental evidence supports the fact that serotonergic psychedelics alter the metabolism and activation levels of the occipital lobe. An inverse correlation between the intensity of the experienced visual imagery and the spectral power of cortical oscillations in the alpha (8–12 Hz) band suggests that visual imagery induced by LSD could relate to a loss of top-down inhibition of ongoing spontaneous activity (Carhart-Harris et al., 2016), given that alpha oscillations have been implicated in the suppression of such activity (Klimesch et al., 2007; Jensen and Mazaheri, 2010). In agreement with these findings on LSD-induced visual imagery, previous studies have also linked alpha suppression to the formation of visual imagery induced by other serotonergic psychedelics such as psilocybin (Kometer et al., 2013) or ayahuasca (Valle et al., 2016). The fact that alpha oscillations inhibit large-range functional connectivity as inferred from functional magnetic resonance recordings (Tagliazucchi et al., 2012; Chang et al., 2013) supports the internal consistency of the multimodal results reported by Carhart-Harris et al. (2016).

More generally, serotonergic psychedelics appear to elicit their effects by disrupting inhibitory processes in the brain (Guilbaud et al., 1973; Haigler and Aghajanian, 1973; Vollenweider et al., 1997; Kometer et al., 2013; Carhart-Harris R. et al., 2014; Nichols, 2016; Schmidt et al., 2017). The serotonergic psychedelic psilocybin has been shown to reduce brain metabolism in the posterior cingulate cortex (PCC), a key hub in the default mode network (DMN) of the brain that could exert an inhibitory influence in other brain regions (Carhart-Harris et al., 2012). It has been hypothesized that the dreamlike quality of the experience elicited by psilocybin relates to increased activity in the medial temporal lobe (MTL) as a result of decreased PCC-mediated inhibition (Carhart-Harris and Nutt, 2014; Carhart-Harris R. et al., 2014). This hypothesis is consistent with neuroimaging studies showing that MTL activity increases after psilocybin infusion (Tagliazucchi et al., 2014) and that such activity increases are correlated to the subjective assessment of the dreamlike quality of the experience (Carhart-Harris and Nutt, 2014). MTL activity is also enhanced in humans during REM sleep (Maquet et al., 1996; Miyauchi et al., 2009) and pathological increases in MTL activity due to temporal lobe epilepsy can result in an altered state of consciousness that is ascribed a dreamlike quality by the patients (Gloor et al., 1982). Suppressed PCC activity also is a landmark feature of human REM sleep (Maquet et al., 1996; Braun et al., 1997), suggesting that loss of PCC-mediated inhibition also underlies altered consciousness during dreaming. Furthermore, the direct electrical stimulation of the PCC has been shown to induce a state that is both similar to the serotonergic psychedelic experience and dreaming (Herbet et al., 2014). This study provides evidence of a causal relationship between disruption of PCC-mediated cortical inhibition and the dreamlike quality of the subjective experience. Further causal evidence is provided by the observation that dreamlike experiences can be elicited by direct electrical stimulation of the MTL (Halgren et al., 1978). Thus, we propose that altered consciousness during the psychedelic experience and dreaming might relate to disrupted PCC and MTL activity, providing a common neurophysiological basis for their phenomenological similarity.

Less is known about the relationship between the changes in brain activity due to dissociative psychedelics and dreaming. These substances act by inhibiting the propagation of brain activity, either by decreasing excitation (e.g., ketamine, an NMDA antagonist; Tyler et al., 2017) or increasing inhibition (e.g., muscimol, a selective GABA_A_ agonist present in *Amanita muscaria* mushrooms; Frølund et al., 2002). As in the case of serotonergic psychedelics, the analysis of magnetoencephalography recordings acquired after the infusion of a sub-anesthetic dose of ketamine show widespread decreases in alpha power. Whether a mechanistic explanation for these changes can be found in the loss of PCC-mediated cortical inhibition remains to be investigated (Muthukumaraswamy et al., 2015).

From a pharmacological perspective, the similarity between dreaming and the effects of deliriant substances is paradoxical, since tropane alkaloids act by blocking the neurotransmitter acetylcholine (i.e., they are anticholinergic agents; Safer and Allen, 1971), whereas dreaming is associated with higher levels of acetylcholine (Hobson, 1992b). The acute deliriant effects of the tropane alkaloids contained in plants such as those in the *Datura* and *Brugmansia* genera are difficult to investigate in humans using neuroimaging tools, and whether their effects depend on the disruption of cortical inhibition remains unknown. However, electroencephalography and magnetoencephalography studies of scopolamine do not show the drop in alpha power that is typical of serotonergic psychedelics and ketamine (Ebert and Kirch, 1998; Osipova et al., 2003), suggesting a different mechanistic explanation for its acute effects.

### Changes in self-awareness

During ordinary wakeful consciousness the first-person point of view is intertwined with the bodily location: the self is located within a body recognized as its own, with well-defined boundaries and a sense of agency over its actions and movements. This perspective can be deeply modified during dreaming and by the effect of psychoactive substances. While frequently the point of view of the dreamer is consistent with a first-person perspective, the dreamer often sees herself and her actions from a third-person perspective (Thompson, 2014). This divergence is also typical after high doses of dissociative psychedelics such as ketamine and PCP (Muetzelfeldt et al., 2008; Wilkins et al., 2011; Morris and Wallach, 2014) and is one of the defining characteristics of out-of-body experiences, i.e., experiences involving the feeling of leaving one's body or perceiving it from the outside (autoscopy) (Blanke et al., 2002, 2004; Blanke and Arzy, 2005; Bünning and Blanke, 2005).

The phenomenology of dreaming also includes the experience of “boundlessness” during which the self is either grown to encompass its surroundings or “dissolves” into them (Windt, 2010). This experience is frequently referred to as “oceanic boundlessness” or “ego-dissolution” (Millière, 2017). Serotonergic psychedelics have long been known to induce ego dissolution experiences, which have been sometimes ascribed a spiritual or mystical character (Strassman, 2000; Griffiths et al., 2006). As Albert Hofmann recalls from his first LSD experience: “Ego and the outer world are separated in the normal condition of consciousness, in everyday reality; one stands face-to-face with the outer world; it has become an object. In the LSD state the boundaries between the experiencing self and the outer world more or less disappear, depending on the depth of the inebriation” (Hofmann, 1980). As recently reviewed by Millière, 7% of Erowid subjective reports of psilocybe mushrooms, LSD, *Salvia divinorum*, DMT, 5-MeO- DMT, ayahuasca and ketamine describe an ego-dissolution experience (Millière, 2017). A higher incidence (≈50%) of ego-dissolution experiences has been reported in large-scale studies using questionnaires (Griffiths et al., 2006; Studerus et al., 2011); this discrepancy is likely related to the use of questionnaires vs. freely reported narratives. Consistent with these observations, ego-dissolution is also reported when LSD and psilocybin are administered experimentally (Lebedev et al., 2015; Carhart-Harris et al., 2016; Tagliazucchi et al., 2016; Liechti et al., 2017).

These observations suggest that changes in self-awareness, related either to the dissociation between the first-person point of view and the bodily self or to the loss of boundaries between the bodily self and the environment, are factors driving the semantic similarity between subjective reports of hallucinogens and dreaming. Also, the prevalence of words representing persons closely related to the narrator (“mom,” “dad,” “brother,” “parent,” “family”) could indicate altered self-referential processing during dreams and under the influence of hallucinogens. Whether both experiences share common neurophysiological bases is to be determined by future experiments. The neural mechanisms of drug-induced ego-dissolution have received more attention than their REM sleep counterparts, presumably due to the difficulty of performing neuroimaging experiments during sleep in combination with the necessity of adopting a serial awakening paradigm to probe the occurrence of distortions in self-awareness. A recent study has shown that LSD-induced ego-dissolution relates to increased global connectivity of fronto-parietal and insular regions, especially those located within the temporo-parietal junction (Tagliazucchi et al., 2016). These results are consistent with the proposition that out-of-body experiences and other alterations in self-awareness stem from a failure to integrate multisensory information at the temporo-parietal junction (Blanke and Arzy, 2005). Future studies should investigate whether this proposition also holds for disturbed self-awareness during REM sleep.

While hallucinations are occasionally reported for drugs chemically unrelated to dissociative/serotonergic psychedelics and deliriants, alterations in self-awareness (including but not limited to those mentioned in the previous paragraphs) seem to be specific to these substances and to the kappa opioid receptor agonist *Salvia divinorum* (Millière, 2017). This plant contains salvinorin-A, a kappa opioid receptor agonist capable of producing potent hallucinogenic effects (Roth et al., 2002). These effects include derealization, depersonalization and detachment from the self and the environment, in combination with intense simple and complex visual imagery, and alterations in auditory and vestibular input (Sumnall et al., 2011; MacLean et al., 2013; Addy et al., 2015). Thus, the effects of *Salvia divinorum* include certain features of serotonergic psychedelics (i.e., the nature of the visual imagery), dissociative psychedelics (depersonalization and derealization) and anticholinergic deliriants (impaired awareness of undergoing a drug-induced experience). These observations are consistent with the high similarity between *Salvia divinorum* reports and dreams (Figures 1, 2), and suggest further experimental work to elucidate the physiological underpinnings of this similarity.

### Changes in metacognition and dream lucidity

As we know from our everyday experience, during most dreams the dreamer is not aware of experiencing a dream and lacks voluntary control over her actions. This lack of awareness occurs in spite of events that would stand out as incongruent, bizarre or outright impossible during wakefulness (Nir and Tononi, 2010). The suppression of the capacity to recognize the abnormal nature of such events could be linked to diminished metacognitive function during REM sleep, i.e., the diminished ability of the dreamer to reflect upon and understand her own thought processes (Kahan and LaBerge, 1994). This suppression of insight and self-reflection is consistent with the deactivation of two regions involved in metacognition: the dorsolateral prefrontal cortex (DLPFC) and the frontopolar cortex (Maquet et al., 1996; Braun et al., 1997). Correlational evidence links these brain regions to metacognition (Fleming and Dolan, 2014) and their magnetic stimulation is known to impair metacognitive function (Rounis et al., 2010; Ryals et al., 2015). Conversely, it has been speculated that lucidity relates to preserved metacognitive capacity during REM sleep dreams (Kahan and LaBerge, 1994). A recent study provided support for this claim by showing that lucid dreamers have greater gray matter volume in the frontopolar cortex as well as higher activation in a thought-monitoring task, both relative to a control group of non-lucid dreamers (Filevich et al., 2015).

As in REM sleep episodes, serotonergic psychedelics decrease the oscillatory activity and functional connectivity of the DLPFC (Muthukumaraswamy et al., 2013). In contrast to non-lucid dreamers, the users remain aware of the nature of their experience and can situate themselves within an altered state different from normal wakefulness. However, both states of consciousness are characterized by increased cognitive bizarreness of mental imagery (Kraehenmann et al., 2017). Cognitive bizarreness can be defined as the presence of improbable, impossible or incongruent events during a given experience, and has been shown to be a reliable indicator of dreamlike mentation (Hobson et al., 1987). Furthermore, Kraehenmann et al. have shown that LSD-induced increases in cognitive bizarreness correlate with other aspects of the psychedelic experience such as loss of self-boundaries and cognitive control, and are mediated by serotonin 5-HT_2A_ receptor activation (Kraehenmann et al., 2017). An important difference between the psychedelic state and non-lucid dreams is that only in the latter the disruption of metacognitive function limits the capacity for reflecting upon the cognitive bizarreness of the experience and determining the departure from ordinary wakefulness, which is possibly related to hypofrontality during sleep (Dietrich, 2003).

Deliriants are known to induce a confusional state during which the user can lose awareness of experiencing drug-induced alterations in consciousness. It is interesting to note that a larger proportion of deliriant substances ranked among the most similar to dream reports of low lucidity in comparison to dreams of high lucidity. In the first case the top three ranking substances included two deliriant agents (plants of the *Datura* and *Brugmansia* genera) while in the second case they included only one (*Daturas*). We must note, however, that no significant differences were found when comparing their similarity to dreams of high vs. low lucidity (Figure 3). Thus, further study is required to assess whether the preservation of metacognitive function during lucid dreams decreases the phenomenological similarity to deliriant-induced alterations in consciousness.

A category of substances highly relevant to the present discussion is that of oneirogens. The Erowid corpus contains reports of two plants primarily classified as oneirogens: *Calea zacatechichi* and *Silene undulata* (Schultes and Hofmann, 1979). *Calea zacatechichi* is a flowering plant native to Mexico and Central America that is consumed, among other uses, for its capacity to induce vivid dreams. It is believed that certain sesquiterpenes underlie its oneirogen effects (Herz and Kumar, 1980). *Silene undulata*, also known as *Silene capensis* or African dream root, is a plant native to South Africa traditionally employed by the Xhosa people to facilitate vivid and lucid dreams (Sobiecki, 2008). While it might be surprising that the subjective reports of these two plants do not rank higher in terms of their similarity to dreaming, the word cloud for *Calea zacatechichi* (Figure 7) indicates that the most frequent terms were related to recommendations, expectations, and the required preparation process of the plant. However, it is interesting to note that the subjective reports associated with *Calea zacatechichi* and *Silene undulata* increased their ranking more than any other substance when compared to dreams of high lucidity vs. dreams of low lucidity. This might relate to the capacity of these plants to restore metacognitive function during sleep, a possibility requiring further scientific investigation.

### Methodological considerations and limitations

We propose that the application of tools for semantic analysis such as LSA to large corpora of subjective experiences could open the way to the development of a quantitative “comparative phenomenology” allowing to determine the phenomenological similarity between altered states of consciousness and to relate such similarity to the underlying neurophysiological mechanisms (Varela, 1996). While understanding the phenomenological subtleties of different experiences requires careful introspection and reporting (Thompson and Zahavi, 2007), the “ecological” approach of analyzing freely written subjective reports is likely sufficient to determine the degree of similarity between altered states of consciousness. Ample literature ascribing dreamlike qualities to certain hallucinogenic substances, together with the availability of large and curated corpora of subjective reports, made the present analysis a natural first choice to apply this methodology. Future analyses should address the hypothesis that certain spontaneously occurring altered states of consciousness (such as near death experiences) are induced by psychedelic compounds endogenous to the human brain (Strassman, 2000).

The large number of subjective reports available from online databases such as Erowid and Dreamjournal are one of the main strengths of our study, but also represent a source of limitations. Even though both databases are curated, there is no certainty that the substances consumed by Erowid users are chemically pure and that their identity is what the users claim in their reports. Also, Erowid reports frequently lack information on the dosage and, when present, it is not possible to corroborate whether such information is accurate. A placebo-controlled study could alleviate expectation issues in the subjective reports; however, it is unlikely that such study could include a large number of different substances and reports. It is also not possible to determine whether Dreamjournal reports are based on REM sleep episodes or on dreamlike imagery occurring at other times of the sleep cycle, such as hypnagogic or hypnopompic imagery consisting of hallucinatory content that lacks the elaborate narratives of REM sleep dreaming (Hobson et al., 2000). To compensate for the possibility of missing or erroneous information, these databases provide a very large number of reports from which a meaningful signal is likely to emerge in spite of uncontrolled sources of noise (Halevy et al., 2009).

### Conclusions

In summary, we have developed a method to determine the similarity between altered states of consciousness based on large corpora of subjective reports and applied it to corroborate the hypothesis that the experiences elicited by certain drugs bear a high resemblance to dreaming. While this hypothesis chiefly concerned serotonergic psychedelics, we found that dissociative psychedelics and deliriants led to reports of experiences more similar to those of dreams. We speculate that this higher similarity could be based on the nature of the distortions in perception and self-awareness elicited by these substances, combined with the impairment of metacognitive function in the case of deliriants. Future studies should focus on these phenomenological features and on establishing a link with the underlying neurophysiological mechanisms.

## Authors contributions

CS and ET analyzed the data. ET designed the study and wrote the paper. EE and FE designed the Erowid experience report collection system and have managed the collection of psychoactive-related experience reports since 1995. FZ contributed to data analysis, data interpretation, and to the creation and curation of a local database of experience reports.

### Conflict of interest statement

The authors declare that the research was conducted in the absence of any commercial or financial relationships that could be construed as a potential conflict of interest.

## Footnotes

^1^The terms “drug” and “substance” are here used interchangeably to refer to psychoactive molecules and the plants and fungi containing them.

^2^We use the term “hallucinogen” to refer to deliriants and both dissociative and serotonergic psychedelics (see Nichols, 2004).
