# Corrigendum: The Experience Elicited by Hallucinogens Presents the Highest Similarity to Dreaming within a Large Database of Psychoactive Substance Reports

**DOI:** 10.3389/fnins.2018.00229

**Published:** 2018-04-11

**Authors:** Camila Sanz, Federico Zamberlan, Earth Erowid, Fire Erowid, Enzo Tagliazucchi

**Affiliations:** ^1^Departamento de Física, Universidad de Buenos Aires, Buenos Aires, Argentina; ^2^Erowid Center, Grass Valley, CA, United States; ^3^Brain and Spine Institute, Paris, France

**Keywords:** dreams, psychedelics, dissociatives, deliriants, hallucinogens, phenomenology, consciousness

We would like to issue a corrigendum concerning the following points. Please note that these corrections are not related to the analysis of the data and the presentation of the results, nor they affect in any way the conclusions of our article. We deeply apologize for any inconvenience caused.

1. The following authors with the affiliations shown below should be added to the author list of the paper, between C. Sanz (first author) and E. Tagliazucchi (last author) in the following order:

- Federico Zamberlan. Affiliation: Departamento de Física, Universidad de Buenos Aires, Argentina. Email: federicozamberlan@hotmail.com
- Earth Erowid. Affiliation: Erowid Center, Grass Valley, CA, USA. Email: research@erowid.org
- Fire Erowid. Affiliation: Erowid Center, Grass Valley, CA, USA. Email: research@erowid.org

Thus, the author list of the article should read as follows:

“C. Sanz, F. Zamberlan, E. Erowid, F. Erowid, E. Tagliazucchi”

The reason for this correction is the re-evaluation of the merits of the contributors that were originally mentioned in the “Acknowledgments” section of the manuscript. After friendly discussions with E. Erowid and F. Erowid, we reached the agreement that their role in the creation, curation and interpretation of the online database, as well as in the ensuing scientific discussion, should be acknowleged with co-authorship. The addition of F. Zamberlan to the author list is based on similar contributions, related to our local version of the database (creation and curation) and on his scientific input, in all cases fundamental for the research conducted.

2. The following sentence should be added in the Materials and Methods” section, “Corpora Selection” sub-section, right before the sentence: “We discarded reports that resulted…”:

“Our research relied upon Erowid's reviewed and edited collection of experience reports, and followed Erowid's terms of use that require researchers to coordinate with Erowid's research team in order to avoid misinterpretations of their data (https://erowid.org/general/about/about_copyrights.shtml).”

The reason for this correction is to encourage future researchers to comply with Erowid's terms of use. Such compliance is necessary to acknowledge the efforts of the Erowid team, and to avoid misinterpretations of the reports, thus improving the overall scientific accuracy of the resulting manuscripts.

3. The “Author contribution statement” section should be changed as follows to reflect the changes in authorship:

“CS and ET analyzed the data. ET designed the study and wrote the paper. EE and FE designed the Erowid experience report collection system and have managed the collection of psychoactive-related experience reports since 1995. FZ contributed to data analysis, data interpretation, and to the creation and curation of a local database of experience reports.”

4. The “Acknowledgments” section should be changed as follows to reflect the changes in authorship:

“ET was supported by a Marie Skłodowska-Curie individual fellowship. FZ was supported by a CONICET doctoral fellowship. We acknowledge insightful discussions with Facundo Carrillo, Mariano Sigman, and Diego Fernandez Slezak. We also thank the curators and contributors of www.erowid.org and www.dreamjournal.net.”

We apologize for these mistakes, which do not change the scientific conclusions of the article in any way. All authors approved this corrigendum.

The original article has been updated.

## Conflict of interest statement

The authors declare that the research was conducted in the absence of any commercial or financial relationships that could be construed as a potential conflict of interest.

